# Rapid mode switching facilitates the growth of *Trichodesmium*: A model analysis

**DOI:** 10.1016/j.isci.2024.109906

**Published:** 2024-05-03

**Authors:** Meng Gao, Jamal Andrews, Gabrielle Armin, Subhendu Chakraborty, Jonathan P. Zehr, Keisuke Inomura

**Affiliations:** 1 Graduate School of Oceanography, University of Rhode Island, Narragansett, RI, USA; 2 Biological and Environmental Sciences Graduate Program, University of Rhode Island, Kingston, RI, USA; 3 Systems Ecology Group, Leibniz Centre for Tropical Marine Research (ZMT), Bremen, Germany; 4 Department of Ocean Sciences, University of California, Santa Cruz, CA, USA

**Keywords:** Molecular modeling, computational molecular modeling, Biological sciences, Microbiology

## Abstract

*Trichodesmium* is one of the dominant dinitrogen (N_2_) fixers in the ocean, influencing global carbon and nitrogen cycles through biochemical reactions. Although its photosynthetic activity fluctuates rapidly, the physiological or ecological advantage of this fluctuation is unclear. We develop a metabolic model of *Trichodesmium* that can perform daytime N_2_ fixation. We examined (1) the effect of the duration of switches between photosynthetic and non-photosynthetic cellular states and (2) the effect of the presence and absence of N_2_ fixation in photosynthetic states. Results show that a rapid switch between photosynthetic and non-photosynthetic states increases *Trichodesmium* growth rates by improving metabolic efficiencies due to an improved balance of C and N metabolism. This provides a strategy for previous paradoxical observations that all *Trichodesmium* cells can contain nitrogenase. This study reveals the importance of fluctuating photosynthetic activity and provides a mechanism for daytime N_2_ fixation that allows *Trichodesmium* to fix N_2_ aerobically in the global ocean.

## Highlights

• O_2_ levels in *Trichodesmium* can decrease rapidly at the start of N_2_ fixation states
• The rapid mode switching allows faster cellular growth than the slower mode
• The faster growth is due to improved C and N allocation in cells
• Our study provides a mechanistic understanding of aerobic daytime N_2_ fixation

## Graphical abstract

**Figure:**
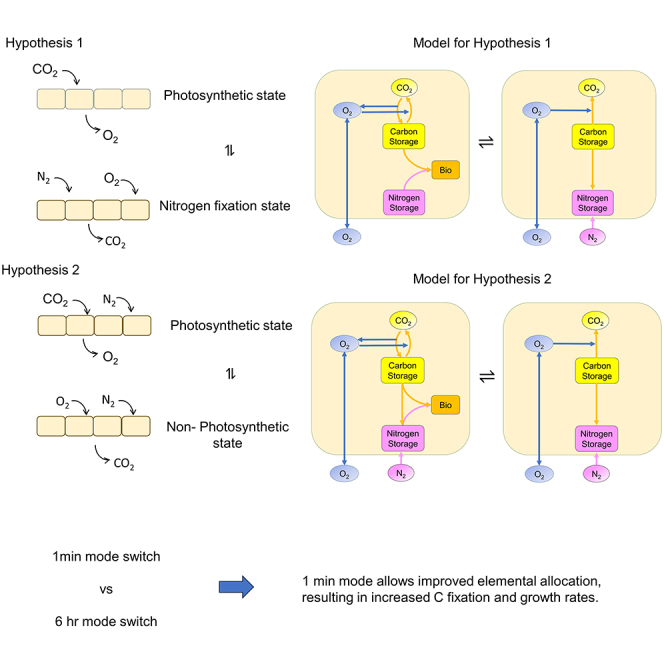

## Introduction

*Trichodesmium* is a cyanobacterial genus whose species have a multicellular filamentous morphology^1^ and are widely distributed in tropical and subtropical areas.^2^^,^^3^^,^^4^^,^^5^^,^^6^^,^^7^ It is also an important dinitrogen(N_2_)-fixing cyanobacterial genus in the global ocean.^8^^,^^9^ In addition to fixing N_2_, *Trichodesmium* is photosynthetic and evolves oxygen (O_2_).^10^^,^^11^ *Trichodesmium* plays an important role in ocean biogeochemical cycles^12^ since it contributes to carbon (C) and nitrogen (N) cycling. Due to its ecological importance, many laboratory experiments^13^^,^^14^^,^^15^ and modeling studies^3^ have been performed to understand its physiology, including N_2_ fixation strategies.^4^^,^^5^^,^^16^

The N_2_-fixing enzyme (nitrogenase) with metal cofactors can be inactivated by presence of O_2_.^17^^,^^18^ Since cyanobacteria evolve O_2_ through oxygenic photosynthesis, they have to use physiological or morphological strategies to avoid the inactivation of nitrogenase.^16^^,^^17^^,^^19^ Several strategies in cyanobacteria have been reported,^17^^,^^19^ for example, the formation of specialized N_2_ fixation cells that lack oxygenic photosynthetic activity (heterocysts).^16^^,^^20^^,^^21^^,^^22^ However, *Trichodesmium* does not form heterocysts.^23^ Although a recent study found that *Trichodesmium* can fix N_2_ in the dark,^24^ many previous studies suggest it appears to fix N_2_ and photosynthesize in the same cells during the daytime with light.^25^^,^^26^^,^^27^^,^^28^ It is still unresolved how *Trichodesmium* fixes N_2_ aerobically in the light while evolving photosynthetic O_2_.

Although *Trichodesmium*’s N_2_ fixation mechanism is still unclear, results of previous studies suggested that photosynthetic activities can be regulated during cellular-level photosystem state transitions, which are on the order of 1 minute.^3^^,^^16^^,^^29^^,^^30^ Based on this photosynthesis 1-min on/off switch, we developed two hypotheses: (H1) nitrogen fixation only occurs during a non-photosynthetic state, or (H2) *Trichodesmium* continues fixing N_2_ during photosynthesis.

As for H1, a model by Inomura et al. 2019^3^ suggested that the intracellular O_2_ may be decreased rapidly on the timescale of seconds without photosynthesis, which provides an opportunity for N_2_ fixation. The rapid recovery of nitrogenase can be supported by evidence from studies in other species, which have a protein (Shethna Protein II, FeSII) for conformational protection of nitrogenase.^31^ This protein can quickly respond to O_2_ and make the nitrogenase in an inactive but oxygen-tolerant state until recovery. However, this protein (or its genes) has not been found in *Trichodesmium*, and reactivation of nitrogenase likely requires a much longer time than seconds to recover from inhibition.^32^^,^^33^ This argument leads to Hypothesis 2 (H2), which states that *Trichodesmium* may continue fixing N_2_ during photosynthesis. Studies show that under ambient oxygen concentrations, it takes more than a few minutes to deactivate nitrogenase.^31^^,^^33^ Thus, *Trichodesmium* might tolerate short time intervals at high O_2_ concentrations, for example, 1 minute. It takes tens of minutes to resynthesize nitrogenase,^34^^,^^35^ which excludes the possibility that cells are constantly resynthesizing nitrogenase if it is damaged; and repair cannot keep pace with inactivation at those short time intervals.

**Figure 1:**
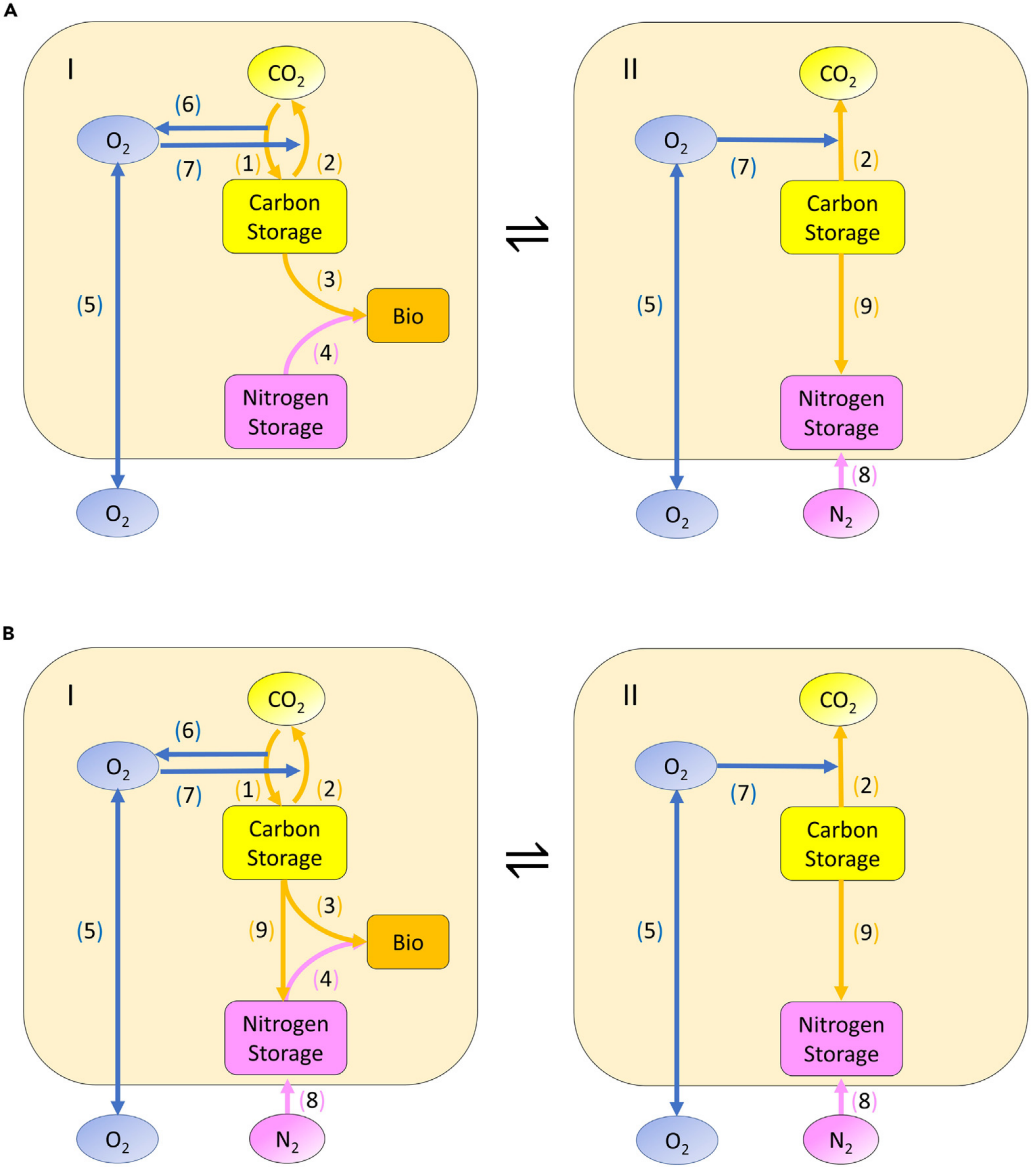
Schematic depiction of molecular pools and fluxes in the model (A) H1: Photosynthesis and N_2_ fixation occur at different times. (B) H2: Photosynthesis and N_2_ fixation occur simultaneously. For (A) and (B): (I) Photosynthetic state. (II) Non-photosynthetic (N_2_ fixation) state. The symbol ⇌ represents state transition. Pathways: (1) C fixation. (2) Respiration. (3) C consumption in growth. (4) N consumption in growth. (5) O_2_ diffusivity. (6) O_2_ production in carbon fixation. (7) O_2_ consumption in respiration. (8) N_2_ fixation. (9) C consumption in N_2_ fixation (for energy and electron). Different colors represent different element pools and fluxes: orange arrows and yellow items are for C, pink items are for N, and blue items are for O_2_. The orange rounded corner rectangles mean biomass. The cream rounded corner rectangles mean *Trichodesmium* cells. According to the previous observation of *Trichodesmium*,^30^ we switched these two states every minute and every 6 hours. We ran the models for 12 hours. And we assumed that there was no growth in the non-photosynthetic state.

Based on these hypotheses, we built a metabolic model of *Trichodesmium* (Figure 1) and ran it under two situations (H1 and H2) and two switch modes (1-min: switch every minute and 6-h: switch every 6 hours) to answer three main questions: (1) How do the cells fix N_2_ even though they are producing O_2_? (2) How can mode switching influence growth rate and why? (3) Based on element (C and N) allocation, which switch mode is more efficient? The following model illustrates the mechanism of *Trichodesmium* N_2_ fixation and the associated advantages of the mechanism.

## Results and discussion

### *Overview of the model*

We developed a physiological model of a *Trichodesmium* cell that performs photosynthesis to obtain carbon (C) and fix N_2_ to obtain nitrogen (N). The cell produces O_2_ during photosynthesis and uses both C and O_2_ to maintain respiration. Based on previous experimental evidence,^30^ here we assume that the cell can switch between photosynthetic and non-photosynthetic states. Accordingly, we built and examined two hypotheses H1 and H2; H1: N_2_ fixation happens only during the non-photosynthetic state (Figure 1A, Video S1), whereas H2: N_2_ fixation happens in both photosynthetic and non-photosynthetic states (Figure 1B, Video S2). Moreover, we considered two different switch modes based on the time duration of each state: a rapid mode, where the switching between two states happens every minute (1-min mode), and a slow mode, where the switching happens every 6 hours (6-h mode). To calculate cellular element dynamics (C, N, O_2_), we resolved molecular transport, photosynthesis, respiration, biosynthesis, and N_2_ fixation as the critical pathways. The following are the most important results.

Video S1. State transition for H1

Video S2. State transition for H2. (In the molecules shown in the Videos S1 and S2, red means O atoms, blue means N atoms, and gray means C atoms. We include O_2_, CO_2_ and N_2_.)

#### O_2_ fluctuation analysis

**Figure 2:**
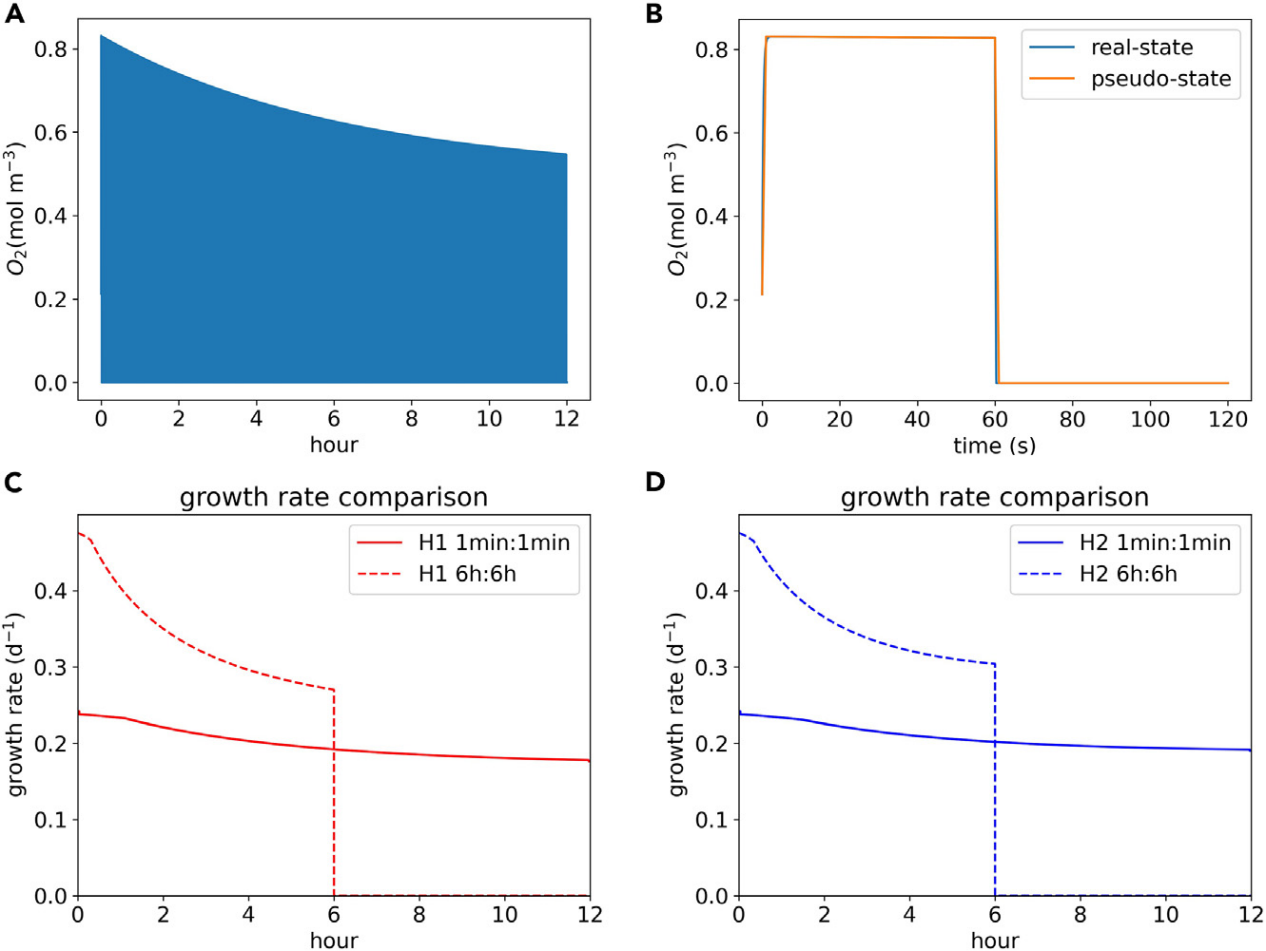
O_2_ level and growth rate changes in 12 h (A) Changes in O_2_ concentration in 12 h (H2, and H1 pattern is similar in Figure S1). (B) Changes in O_2_ concentration in 2 min (H2, and H1 pattern is similar in Figure S1); 60 s for photosynthetic state and 60 s for non-photosynthetic state. Pseudo-state means we used steady-state conditions (O_2_ values do not change) for the simulation. (C) Growth rate comparison of different modes in H1. (D) Growth rate comparison of different modes in H2. For (C) and (D), in the rapid switch mode, we plot the growth rate by taking an average of 2 min.

Our results show that O_2_ concentrations change dramatically as the state switches in both hypotheses. Figure 2A and 2B show that it took less than 1 s during the daytime to reach a steady O_2_ concentration after switching to the non-photosynthetic (N_2_ fixation) state. In the photosynthetic state, the O_2_ reached a high level, while in the N_2_ fixation state, O_2_ concentration decreased and remained low. The rapid decrease of O_2_ in the N_2_ fixation state might provide conditions to allow nitrogenase activity. Nitrogenase might (H1) reactivate during low O_2_ conditions or (H2) tolerate the high O_2_ level for a short time during the photosynthetic state. Based on these rapid O_2_ changes, *Trichodesmium* can photosynthesize and fix nitrogen (Figure S2) in the same cell, even with the temporal separation of only 1 minute.

Our simulated quick changes in O_2_ concentrations are similar to those of a previous modeling study,^3^ which estimated an extremely short residence time of O_2_ (the time to consume all O_2_ by respiration) in *Trichodesmium* cells on the order of 1 s. The rapid decrease results from the high respiration rate, as suggested by previous studies.^3^^,^^23^^,^^36^^,^^37^ Since N_2_ fixation requires substantial energy in the form of ATP (16 ATP per N_2_ fixed), it needs to be coupled with high aerobic respiration rates to provide ATP. The high aerobic respiration rates could explain our results that the intracellular O_2_ level changes rapidly, and that the O_2_ concentration is low throughout the N_2_ fixation state. In *Trichodesmium*, increased respiration in a cell might also reduce the plastoquinone pool and transmit negative signals to photosystem II (PSII), which would decrease photosynthesis and consequently the production of O_2_.^10^^,^^36^ In addition to respiration, previous studies of N_2_-fixers suggested that lower O_2_ levels can also be maintained in the cell^37^^,^^38^ by lowering O_2_ diffusivity^3^ with the use of multiple membrane layers (gram-negative bacterium)^39^ and extracellular polymeric substances (EPS)^40^^,^^41^^,^^42^^,^^43^^,^^44^ (*Azotobacter vinelandii* and *Trichodesmium*), as well as using an alternative electronic transfer (AET) pathway (*Trichodesmium*).^36^

#### Growth rate comparison

We compared growth rates in 12 h of daytime (with light) between rapid mode switching and slow mode switching (Figure 2C and 2D). For both H1 and H2, the average growth rates over 12 h for rapid mode (H1: 0.20 day^−1^, H2: 0.21 day^−1^, hereafter we use d^−1^ to represent day^−1^) are higher than those of slow mode (H1: 0.17 d^−1^, H2: 0.18 d^−1^). Figure 2C and 2D show that under H1 and H2, the first 6-h growth for the rapid mode is lower, but the remaining 6-h growth rate is higher. The difference between these two modes suggests that the rapid switch with higher average growth rates is a better strategy for *Trichodesmium*, which results from a better element supply and distribution inside the cell. To determine the reason for the higher growth rate, we simulated element fate.

#### Element fate and comparison

**Figure 3:**
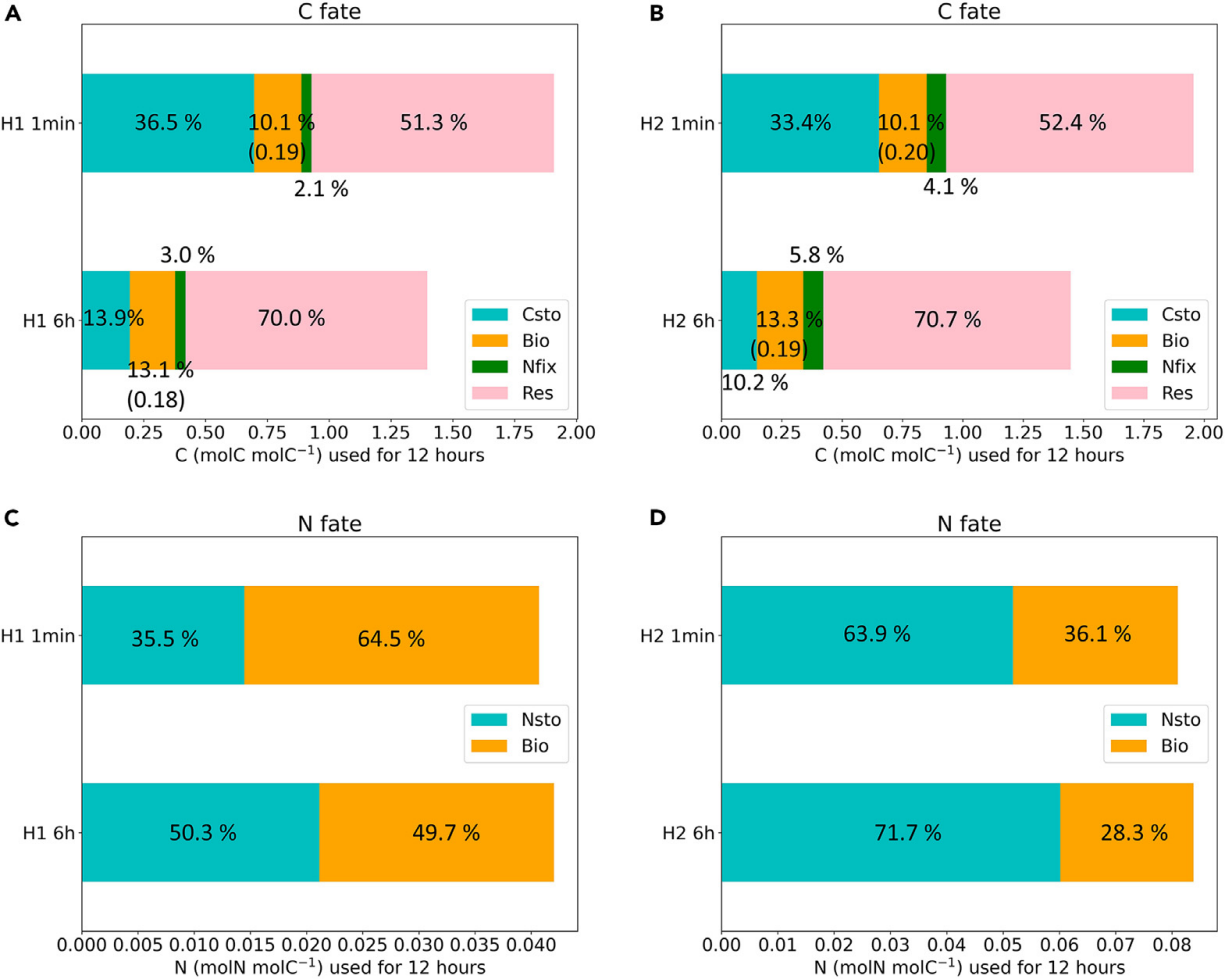
Element fate C fate (C usage distribution in 12 h) comparison for H1 (A) and H2 (B). N fate (N usage distribution in 12 h) comparison for H1 (C) and H2 (D). Csto means C storage, Bio means element (C or N) used in growth, Nfix means C used in N_2_ fixation, Res means C used in respiration, and Nsto means N storage, values in figures mean percentage values, and values in parentheses and the stack length mean the absolute values.

Here we compared C and N fates for two switching modes (Figure 3). During the rapid mode, under H1 and H2, the cell used a larger percentage of C in storage and less in respiration (Figure 3A and 3B). This indicates that in the rapid mode, less percentage of C can be released with respiration, but more C can stay in the cells for metabolisms. Regarding absolute values (indicated in x-labels), the total amount of C invested in cellular processes (including growth) is higher in the rapid mode irrespective of H1 and H2. As for the N fate, results for both hypotheses showed that the rapid mode utilized more N in growth, in terms of both percentage and absolute values, than N storage (Figure 3C and 3D). The total utilization of N for the two modes is slightly different (H1: 0.041 and 0.042, H2: 0.081 and 0.084, unit: mol N mol C^−1^).

We can explain our growth rate results based on the element comparison. To maintain the higher growth rate during the rapid mode switching, more N is used for growth (biomass) and less C percentage is used for respiration. This shows that the rapid mode switching is a more efficient strategy with faster growth and less C lost. Thus, *Trichodesmium* evolved the rapid mode switching to improve benefits from photosynthesis and N_2_ fixation.

In summary, *Trichodesmium* can switch every minute between photosynthesis and N_2_ fixation to achieve temporal separation. This can happen because the O_2_ levels change so fast that nitrogenase can be protected during the N_2_ fixation state. The rapid mode change increases the growth rate by improving the distribution of C and N usage in cells and makes *Trichodesmium* a very successful N_2_-fixer and primary producer in the ocean and dominates the phytoplankton community in some areas.^5^^,^^45^^,^^46^^,^^47^^,^^48^^,^^49^

#### Comparison to previous studies and implications for future work

Previous research suggested that both spatial and temporal separation mechanisms were used in *Trichodesmium*. Studies reported that *Trichodesmium* could form specialized cells (termed diazocytes) where N_2_ fixation was localized.^15^ However, it is still controversial since several studies reported that nitrogenase is randomly distributed in *Trichodesmium* cells or even in all cells, and a modeling study found that spatial separation is unnecessary.^36^ As a result, temporal separation is thought to be more necessary. Rapid state transition switches were shown in cellular-level fluorescence kinetics experiments.^29^^,^^30^ Our study reconciles these observations and provides possible mechanisms that facilitate daytime N_2_ fixation. Our study not only elucidates *Trichodesmium*’s O_2_ protection mechanisms but also reveals a strategy for non-heterocyst forming and daytime N_2_-fixing cyanobacteria, which can be a model for mechanisms in other species in addition to *Trichodesmium*. This model may provide a metabolic module of *Trichodesmium* for existing ecological models with *Trichodesmium*^50^^,^^51^^,^^52^ for predicting their physiological response to the environment and the consequent ecological and biogeochemical impacts.

In our results, rapid mode switching results in higher efficiency in terms of resource use with more N in growth and less C percentage in respiration compared to slow mode switching. Our simulated growth rates are consistent with experimentally observed *Trichodesmium* growth rates (0.1–0.5 d^−1^) in previous studies.^3^^,^^53^^,^^54^ In addition, our simulated nitrogen fixation rates (Figure S2) are in a reasonable scale compared to previous observations (0.006–0.569 mol N mol C^−1^ d^−1^).^11^^,^^55^^,^^56^ Comparing our simulated average N_2_ fixation rate and growth rate data with previous studies results (Figure S3), we found that our model is reasonable, and our results are similar with the real condition. Interestingly, nitrogenase can also be regulated by several environmental factors, and thus environmental factors may be important in affecting *Trichodesmium* N_2_ fixation and growth in the ocean. For example, ocean acidification^57^^,^^58^ and nutrients like iron^57^^,^^59^ have been found to influence nitrogenase efficiency. Future research on how environmental factors (e.g., ocean pH, and nutrients like iron and phosphorus) affect nitrogenase and state transitions will be explored, which is important for predicting the response of *Trichodesmium* to climate change.

### Limitations of the study

The simulated results of the model show the possibility and advantages of the rapid switching mechanisms. However, more experimental studies and observations are needed to show more evidence. In this study, we set a simplified average initial condition and have not considered how different environmental conditions influence the state transition, growth rate, and element allocation. Potential further studies can focus on highly different nutrient conditions (e.g., N or P limitation) that can influence the metabolisms. Although our model is general, some necessary changes will be required for applying it to other N_2_ fixers, e.g., for heterocyst N_2_ fixers like *Rachelia*, which involves different controlling mechanisms for O_2_. Additionally, adjustments to parameters will also be necessary when applying this model to other organisms.

### Conclusion

With a mechanistic model of *Trichodesmium*, we found that rapid mode switching could facilitate N_2_ fixation because cellular O_2_ concentrations can decrease to a very low level in 1 second. This switch mode can also explain why most *Trichodesmium* cells contain nitrogenase and how they can fix N_2_ during the day. The rapid mode switching also keeps C and N concentrations flexible in cells to improve C and N allocation, thus facilitating the growth of *Trichodesmium*. Our results show that switching mode rapidly is an efficient strategy for the growth of non-heterocyst-forming cyanobacteria and daytime N_2_-fixers, which can guide further study in ocean N_2_-fixers.

## STAR★Methods

### Key resources table

**Table:** 

REAGENT or RESOURCE	SOURCE	IDENTIFIER
**Deposited data**		

Simulated data	This paper	[Tricho_Gao_2024]: https://doi.org/10.5281/zenodo.8062145
Experimental test data	Inomura et al. 2020^60^	https://doi.org/10.3390/plants9020192

**Software and algorithms**		

Python code for the model	This paper	[Tricho_Gao_2024]: https://doi.org/10.5281/zenodo.8062145

### Resource availability

#### Lead contact

Further information and requests for resources and reagents should be directed to and will be fulfilled by the lead contact, Meng Gao (meng_gao@uri.edu).

#### Materials availability

This study did not generate new unique reagents.

#### Data and code availability

• All the simulated and experimental data have been deposited at [Tricho_Gao_2024] and are publicly available as of the date of publication. DOIs are listed in the key resources table.
• All original code has been deposited at [Tricho_Gao_2024] and is publicly available as of the date of publication. DOIs are listed in the key resources table.
• “Any additional information required to reanalyze the data reported in this work paper is available from the lead contact upon request.”

### Method details

Here, we describe the ecological model and calculation in detail. Our model includes the photosynthetic state and non-photosynthetic (N_2_ fixation) state of marine phytoplankton *Trichodesmium* (Figure 1). We calculated cellular C, N, and O_2_ concentrations during the half of the diurnal cycle using diffusivity, photosynthesis, respiration, N_2_ fixation, and biosynthesis. Based on our two hypotheses, photosynthesis and N_2_ fixation can happen at the same time in H2, whereas they are separated by time in H1. In the following, first, we describe photosynthetic states for the two hypotheses and then the N_2_ fixation states. The following are the equations we used to build the model.

#### Photosynthetic state

*H1*. To model the phytoplankton C changing rate ($\frac{dC_{sto}}{dt}$) (Equation 1) in the photosynthetic state, we assumed that it could be described as the difference between the C fixing rate ($F_{cfix}$) and C consuming rate. This consumption includes respiration ($F_{Bio}$ E) and growth ($F_{Bio}$), where E is the ratio of respiration to biomass production.

(Equation 1) $$\frac{dC_{sto}}{dt}=F_{cfix}-F_{Bio}\left(1+E\right)\text{.}$$

To estimate cellular O_2_ flux ($\frac{d\left[O_{2}\right]}{dt}$), we included diffusivity, photosynthesis, and respiration pathways (Equation 2). We used a product of diffusivity coefficient (A) and O_2_ difference (${\left[O_{2}\right]}_{E}-\left[O_{2}\right]$) to represent the O_2_ change in diffusivity between the extracellular (E) and intracellular environment. $\rho_{C}^{Bio}$ and $Y_{Cfix}^{O_{2}:C}$ are cell carbon density and O_2_ to C ratio in photosynthesis. $Y_{\mathrm{res}}^{O_{2}:C}$ mean O_2_ to C ratio in respiration. Here we used them to quantify the O_2_ change in carbon fixation ($F_{cfix}$) and respiration ($F_{Bio}E$).

(Equation 2) $$\frac{d\left[O_{2}\right]}{dt}=A\left({\left[O_{2}\right]}_{E}-\left[O_{2}\right]\right)+F_{cfix}\rho_{C}^{Bio}Y_{Cfix}^{O_{2}:C}-F_{Bio}E\rho_{C}^{Bio}Y_{\mathrm{Res}}^{O_{2}:C}\text{.}$$

Next, we calculated N flux ($\frac{dN_{sto}}{dt}$) (Equation 3) which only changes due to the consumption of nitrogen in growth in the photosynthetic state of *Trichodesmium*. Here, we used growth ($F_{Bio}$) times the N to C ratio in cells ($\frac{\left(Y_{Bio}^{N:C}+N_{sto}\right)}{\left(1+C_{sto}\right)}$). In this term, $Y_{Bio}^{N:C}$ represents N to C ratio in biomass. We can also describe it as the N concentration when we use mol N mol C^−1^ as the unit of N content. We added the amount of N in the storage, $N_{sto}$, to it to calculate the whole N concentration ($Y_{Bio}^{N:C}+N_{sto}$). Here we used $\left(1+C_{sto}\right)$ to represent the total C concentration in the cell. 1 here means the original functional C in cells and $C_{sto}$ means C storage in cells.

(Equation 3) $$\frac{dN_{sto}}{dt}=-F_{Bio}\frac{\left(Y_{Bio}^{N:C}+N_{sto}\right)}{\left(1+C_{sto}\right)}\text{.}$$

In Equation 4, we assumed that the C fixation rate ($F_{cfix}$) could increase with light intensity (*I*) but becomes saturated when it reaches a maximum ($F_{Cfix}^{\max}$). Here, $A_{i}$ represents the light saturation coefficient.

(Equation 4) $$F_{Cfix}=F_{Cfix}^{\max}\left(1-e^{-A_{i}I}\right)\text{.}$$

In Equation 5, we considered C and N cellular concentrations as two factors limiting biomass production. Due to Liebig’s law of minimum, growth is determined by the scarcest factors. Besides, biomass production can increase with C and N concentrations but will reach saturation at a high value. As a result, the form of the equation resembles Monod kinetics. Therefore, we calculated the actual biomass production, $F_{Bio}$, using the minimum of available C ($\frac{C_{sto}}{C_{sto}+K_{C}}$), and N ($\frac{N_{sto}}{N_{sto}+K_{N}}$) for biosynthesis. Here, $K_{C}$ means the half-saturation concentration for C and $K_{N}$ means the half-saturation concentration for N.

(Equation 5) $$F_{Bio}=F_{Bio}^{\max}\;\min\left(\frac{C_{sto}}{C_{sto}+K_{C}},\frac{N_{sto}}{N_{sto}+K_{N}}\right)$$

In Equation 6, we calculated growth rates (μ) based on biomass production ($F_{Bio}$). And we divide it by (1 + $C_{sto}$), which means total C in cells to transfer the unit. This growth rate equation can be used for all the states we discuss here.

(Equation 6) $$\mu =\frac{F_{Bio}}{1+C_{sto}}$$

*H2.* Here N_2_ fixation happens during photosynthesis, and thus we modified Equations 1, 2, and 3 accordingly:

To calculate the C changing rate, we added N_2_ fixation as another source of expenditure of C (Equation 7). We included two more terms: $F_{N_{2}fix}Y_{N_{2}fix}^{C:N}$ and $F_{N_{2}fix}Y_{N_{2}fix}^{N:O_{2}}Y_{Res}^{C:O_{2}}$, which respectively mean the C usage in N_2_ fixation and the C usage in respiration of N_2_ fixation. Here, $F_{N_{2}fix}$ means N fixation rate, $Y_{N_{2}fix}^{C:N}$ represents C to N ratio in N_2_ fixation, $Y_{N_{2}fix}^{N:O_{2}}$ represents a conversion factor from N to O_2_ in N_2_ fixation, and $Y_{Res}^{C:O_{2}}$ means C to O_2_ ratio in respiration.

(Equation 7) $$\frac{dC_{sto}}{dt}=F_{cfix}-F_{Bio}\left(1+E\right)-F_{N_{2}fix}Y_{N_{2}fix}^{C:N}-F_{N_{2}fix}Y_{N_{2}fix}^{N:O_{2}}Y_{Res}^{C:O_{2}}$$

To revise the O_2_ flux equation, we subtracted a term ($F_{N_{2}fix}\rho_{C}^{Bio}Y_{N_{2}fix}^{N:O_{2}}$) representing the respiratory cost of O_2_ during N_2_ fixation (Equation 8).

(Equation 8) $$\frac{d\left[O_{2}\right]}{dt}=A\left({\left[O_{2}\right]}_{E}-\left[O_{2}\right]\right)+F_{cfix}\rho_{C}^{Bio}Y_{Cfix}^{O_{2}:C}-F_{Bio}E\rho_{C}^{Bio}Y_{Res}^{O_{2}:C}-F_{N_{2}fix}\rho_{C}^{Bio}Y_{N_{2}fix}^{N:O_{2}}$$

To revise the N flux equation, we added a N_2_ fixation term ($F_{N_{2}fix}^{\max}\left(\frac{C_{sto}}{C_{sto}+K_{C}}\right)$) (Equation 9), which depends on cellular C storage ($C_{sto}$). We assume that N_2_ fixation increases with increasing C storage and saturates due to physiological constraints to the maximum limit of N_2_ fixation rate ($F_{N_{2}fix}^{\max})$.

(Equation 9) $$\frac{dN_{sto}}{dt}=-F_{Bio}\frac{\left(Y_{Bio}^{N:C}+N_{sto}\right)}{\left(1+C_{sto}\right)}+F_{N_{2}fix}^{\max}\left(\frac{C_{sto}}{C_{sto}+K_{C}}\right)$$

#### Non-photosynthetic (N_2_ fixation) state

This state is the same under both the hypotheses H1 and H2. To calculate C changing rate in the N_2_ fixation state, we assumed that it could be affected by two pathways: N_2_ fixation ($F_{N_{2}fix}Y_{N_{2}fix}^{C:N}$) and respiration ($F_{Res}/Y_{Res}^{O_{2}:C}$). Here, $F_{N_{2}fix}$ and $F_{Res}$ represent N_2_ fixation rate and respiration rate, respectively. We use C to N ratio ($Y_{N_{2}fix}^{C:N}$ ) in N_2_ fixation and O_2_ to C ratio ($Y_{Res}^{O_{2}:C}$) in respiration to transform the units.

(Equation 10) $$\frac{dC_{sto}}{dt}=-F_{N_{2}fix}Y_{N_{2}fix}^{C:N}-F_{Res}/(Y_{Res}^{O_{2}:C}\rho_{C}^{Bio})$$

We calculate respiration rate ($F_{Res}$) in Equation 11 by assuming that it increases with cellular O_2_ concentration ($\left[O_{2}\right]$) and can reach a maximum rate ($F_{Res}^{\max}$).

(Equation 11) $$F_{Res}=F_{Res}^{\max}\left(\frac{\left[O_{2}\right]}{\left[O_{2}\right]+K_{O_{2}}}\right)$$

Here, $K_{O_{2}}$ means half-saturation concentration of O_2_.

The only pathway affecting the N changing rate ($\frac{dN_{sto}}{dt}$) is N_2_ fixation ($F_{N_{2}fix}$), which is calculated in Equation 12. N_2_ fixation depends on cellular C storage ($C_{sto}$). We assume that N_2_ fixation increases with increasing C storage and saturates due to physiological constraints to the maximum limit of N_2_ fixation rate ($F_{N_{2}fix}^{\max})$ as:

(Equation 12) $$\frac{dN_{sto}}{dt}=F_{N_{2}fix}=F_{N_{2}fix}^{\max}\left(\frac{C_{sto}}{C_{sto}+K_{C}}\right)\text{,}$$

where $K_{C}$ is the half-saturation constant of C storage.

The cellular O_2_ flux (Equation 13) is obtained by subtracting the respiratory consumption from the diffusive O_2_ input:

(Equation 13) $$\frac{d\left[O_{2}\right]}{dt}=A\left({\left[O_{2}\right]}_{E}-\left[O_{2}\right]\right)-F_{Res}^{\max}\left(\frac{\left[O_{2}\right]}{\left[O_{2}\right]+K_{O_{2}}}\right)$$

We calculated all of the element dynamics in the two states by simplification of the Taylor Expansion, expressed by:

(Equation 14) $$Con\left(t+\Delta t\right)=Con\left(t\right)+\frac{dCon}{dt}\Delta t$$

where Con is the concentration of C, N, or O_2_ and $\frac{dCon}{dt}$ is the flux.

#### Element fate calculation

We used Equation 15 to calculate element usage. Here, ${Con}_{i}^{j}$ represents element *i* (includes C and N) usage concentration in *j* pathway (storage, respiration, biomass, N_2_ fixation). We calculated it by summing up the element usage in every time step, which is calculated by $F_{i}^{j}$ (the changing rate of element *i* in *j* pathway, e.g., C storage rate, C changing rate in respiration, C changing rate in growth) multiplied by the time step Δt.

(Equation 15) $${Con}_{i}^{j}=\sum \left(F_{i}^{j}\times \Delta t\right)$$

Equation 16 represents the calculation of the percentage of element usage. Here, ${Per}_{i}^{j}$ represents the percentage of *j* pathway (storage, respiration, biomass, N_2_ fixation) of element *i* (includes C and N) usage. ${Con}_{i}^{Total}$ means the total element *i* usage.

(Equation 16) $${Per}_{i}^{j}=\frac{{Con}_{i}^{j}}{{Con}_{i}^{Total}}\times 100\%$$

#### Model simulation

In this study, we did four simulations. We simulated the model under H1 and H2, and under each hypothesis, we simulated two state switching conditions: a 1-min switch and a 6-h switch. All of these happened in 12 h of daytime with light exposure.

#### Parameters

All parameters we used are included in the supplementary material (Table S1). Most required parameters are obtained or adapted from a previous *Trichodesmium* modeling study,^3^ including initial elemental concentrations, environmental O_2_ levels, half saturation concentrations, light intensity and coefficient, elemental ratio in biochemical reactions, maximal photosynthesis, N_2_ fixation and growth rates, etc. We show these parameters in detail in Table S1.

### Quantification and statistical analysis

O_2_ concentrations dynamics (Figures 2A and 2B) were calculated from Equations 8 and growth rates (Figures 2C and D) were calculated from Equation 6. The element allocation comparison (Figure 3) was calculated from Equations 15 and 16. All the simulations and calculations were completed by using Python 3.8.8 in Eclipse.
