# Supplementary material for: Rapid mode switching facilitates the growth of Trichodesmium: A model analysis
Source: iScience. 2024 May 3;27(6):109906. doi: 10.1016/j.isci.2024.109906 (PMC11214483; doi:10.1016/j.isci.2024.109906)
Supplement: Document S1. Figures S1–S3 and Table S1 [file mmc1.pdf]

iScience, Volume *27*

Supplemental information

Rapid mode switching facilitates the growth of *Trichodesmium*: A model analysis

Meng Gao, Jamal Andrews, Gabrielle Armin, Subhendu Chakraborty, Jonathan P. Zehr, and Keisuke Inomura

Table S1. Parameters and values. (Related to STAR★Methods, Parameters section)

Parameter	Unit	Defination	Value
C_{sto}	mol C mol C^{-1}	Carbon storage	Initial 0.5
F_{cfix}	d^{-1}	Carbon fixation rate	
F_{Bio}	d^{-1}	C changing rate in biomass (growth)	
E	dimensionless unit	The ratio of respiration to biosynthesis	0.4 [S1,S2]
F_{Cfix}^{max}	d^{-1}	Maximum carbon fixation rate	
A_i	μ mol^{-1} m^{2} s	Light saturation coefficient	0.01 [S1]
I	μ mol m^{-2} s^{-1}	Light intensity	700
F_{Bio}^{max}	d^{-1}	Maximum biomass production rate	1 [S1]
K_C	mol C mol C^{-1}	Half saturation constant of C storage	0.2 [S1]
K_N	mol N mol C^{-1}	Half saturation constant of N storage	0.0318 [S1]
$[O_2]$	mol O_2 m^{-3}	Cellular oxygen concentration	Initial 0.213 [S1]
$[O_2]_E$	mol O_2 m^{-3}	Environmental oxygen concentration	0.213 [S3]
ρ_C^{Bio}	mol C m^{-3}	cellular C density	18333 [S4]
$Y_{Cfix}^{O_2:C}$	mol O_2 mol C^{-1}	O_2:C in photosynthesis	1
N_{sto}	mol N mol C^{-1}	Nitrogen Storage	Initial 0.1
A	d^{-1}	Diffusion coefficient of oxygen through cell membrane layers	311,040 [S1]

$\frac{dC_{sto}}{dt}$	d^{-1}	C storage changing rate	
$\frac{d[O_2]}{dt}$	mol O_2 m^{-3} d^{-1}	O_2 concentration changing rate	
$\frac{dN_{sto}}{dt}$	d^{-1}	N storage changing rate	
$Y_{Bio}^{N:C}$	mol N mol C^{-1}	The ratio of N to C in biomass	0.159 [S5]
F_{N_2fix}	mol N mol C^{-1} d^{-1}	N_2 fixation rate	
$F_{N_2fix}^{max}$	mol N mol C^{-1} d^{-1}	Maximum N_2 fixation rate	0.2 [S1]
$Y_{N_2fix}^{C:N}$	mol C mol N^{-1}	The ratio of C to N in N_2 fixation	1 [S6,S7]
$Y_{N_2fix}^{N:O_2}$	mol O_2 mol N^{-1}	Conversion factor from N to O_2 in respiration for N_2 fixation	1.04 [S1]
$Y_{Res}^{O_2:C}$	mol O_2 mol C^{-1}	The ratio of O_2 to C in respiration	1 [S2]
$Y_{Res}^{C:O_2}$	mol C^{-1} mol O_2	The ratio of C to O_2 in respiration	1 [S2]
F_{Res}	mol O_2 m^{-3} d^{-1}	Respiration rate	
F_{Res}^{max}	mol O_2 m^{-3} d^{-1}	Maximum respiration rate	183,330 [S1]
K_{O_2}	mol O_2 m^{-3}	Half saturation concentration of O_2	2×10^{-5}
μ	d^{-1}	Growth rate	

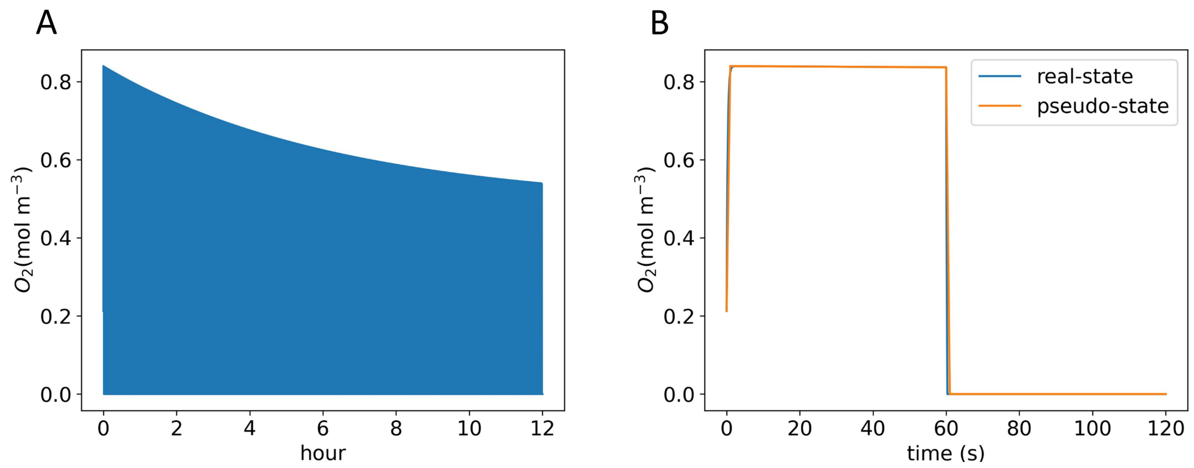

Figure S1. O_2 level and growth rate changes in 12 hours for H1. Related to Figure 2. A.) Changes in O_2 concentration in 12 hours. B.) Changes in O_2 concentration in 2 minutes; 60 s for photosynthetic state and 60 s for non-photosynthetic state.

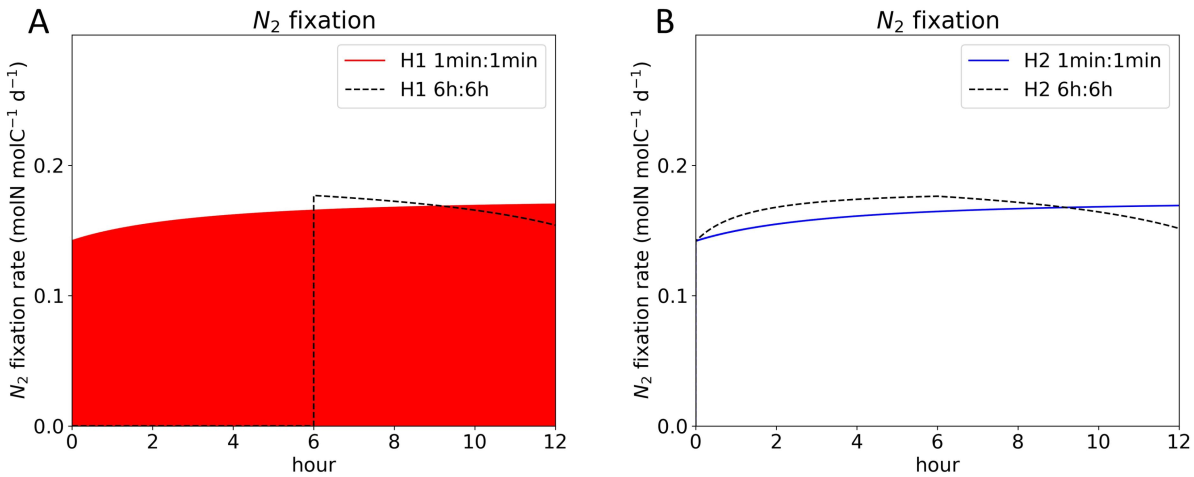

Figure S2. N_2 fixation rates in 12 hours. Related to Figure 2. A.) N_2 fixation rates for different modes under H1. B.) N_2 fixation rates for different modes under H2.

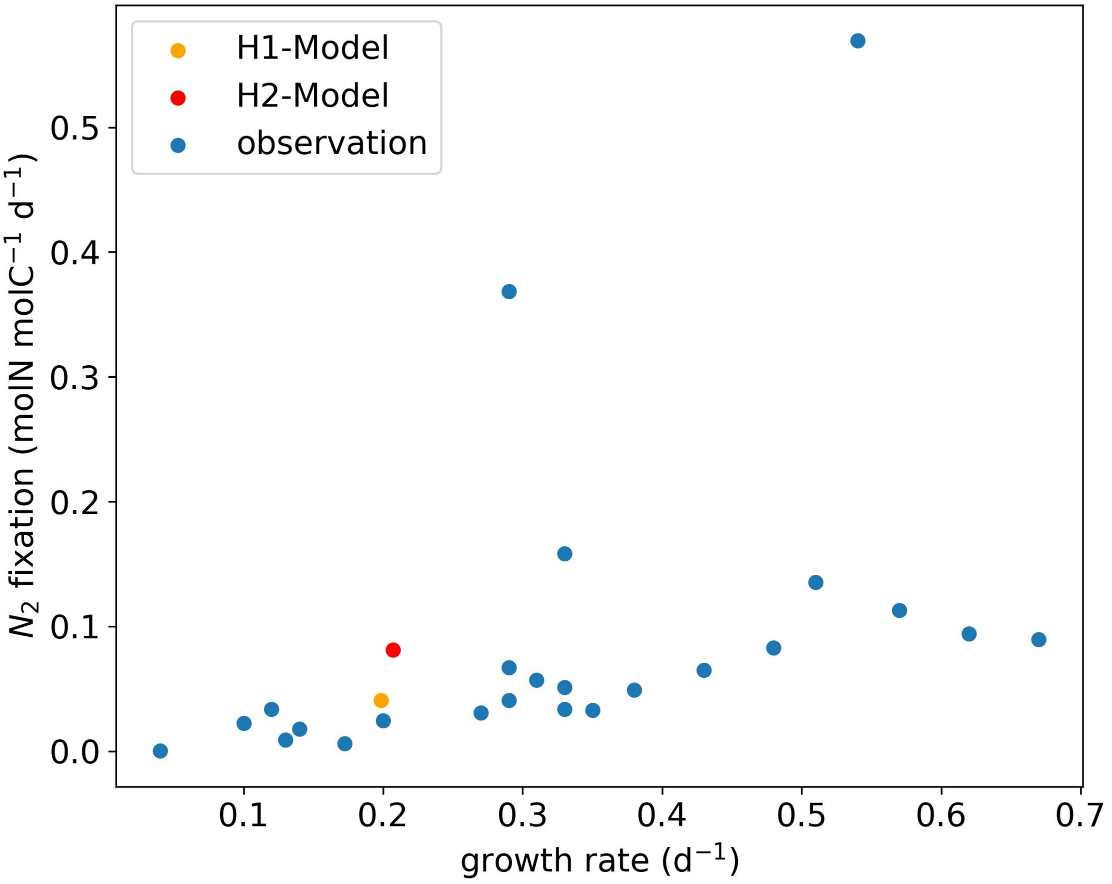

Figure S3. Comparison between model data (rapid mode) and experimental data. Related to Blue dots are experimental data from *Trichodemium* observations [S8–S10] . The orange dot is the simulated average growth rate (for a whole day) and average N_2 fixation in H1. The red dot is the simulated average growth rate (for a whole day) and average N_2 fixation in H2.

References

S1. Inomura, K., Wilson, S.T., and Deutsch, C. (2019). Mechanistic model for the coexistence of nitrogen fixation and photosynthesis in marine *Trichodesmium*. mSystems *4*, 1–13.

S2. Rittmann, B.E., and McCarty, P.L. (2001). Environmental biotechnology: principles and applications (McGraw-Hill Education).

S3. Benson, B.B., and Krause, D. (1984). The concentration and isotopic fractionation of oxygen dissolved in freshwater and seawater in equilibrium with the atmosphere. Limnol Oceanogr *29*, 620–632. 10.4319/lo.1984.29.3.0620.

S4. Bratbak, G., and Dundas, I. (1984). Bacterial dry matter content and biomass estimations. Appl Environ Microbiol *48*, 755–757.

S5. LaRoche, J., and Breitbarth, E. (2005). Importance of the diazotrophs as a source of new nitrogen in the ocean. J Sea Res *53*, 67–91. 10.1016/j.seares.2004.05.005.

S6. Inomura, K., Bragg, J., and Follows, M.J. (2017). A quantitative analysis of the direct and indirect costs of nitrogen fixation: A model based on *Azotobacter vinelandii*. ISME Journal *11*, 166–175. 10.1038/ismej.2016.97.

S7. Inomura, K., Bragg, J., Riemann, L., and Follows, M.J. (2018). A quantitative model of nitrogen fixation in the presence of ammonium. PLoS One *13*, 1–16. 10.1371/journal.pone.0208282.

S8. Holl, C.M., and Montoya, J.P. (2008). Diazotrophic growth of the marine cyanobacterium *Trichodesmium* IMS101 in continuous culture: Effects of growth rate on N_2-fixation rate, biomass, and C:N:P stoichiometry. J Phycol *44*, 929–937. 10.1111/j.1529-8817.2008.00534.x.

S9. Hutchins, D.A., Fu, F.X., Zhang, Y., Warner, M.E., Feng, Y., Portune, K., Bernhardt, P.W., and Mulholland, M.R. (2007). CO_2control of *Trichodesmium* N_2 fixation, photosynthesis, growth rates, and elemental ratios: Implications for past, present, and future ocean biogeochemistry. Limnol Oceanogr *52*, 1293–1304. 10.4319/lo.2007.52.4.1293.

S10. Mulholland, M.R., and Bernhardt, P.W. (2005). The effect of growth rate, phosphorus concentration, and temperature on N_2 fixation, carbon fixation, and nitrogen release in continuous cultures of *Trichodesmium* IMS101. Limnol Oceanogr *50*, 839–849. 10.4319/lo.2005.50.3.0839.
